# Gut Microbiota in Psoriasis

**DOI:** 10.3390/nu14142970

**Published:** 2022-07-20

**Authors:** Mihaela Cristina Buhaș, Laura Ioana Gavrilaș, Rareș Candrea, Adrian Cătinean, Andrei Mocan, Doina Miere, Alexandru Tătaru

**Affiliations:** 1 Department of Dermatology, “Iuliu Hatieganu” University of Medicine and Pharmacy, 400423 Cluj-Napoca, Romania; drmihaelabuhas@gmail.com (M.C.B.); dr.tataru@yahoo.com (A.T.); 2 Department of Bromatology, Hygiene, Nutrition, “Iuliu Hatieganu” University of Medicine and Pharmacy, 23 Marinescu Street, 400337 Cluj-Napoca, Romania; dmiere@umfcluj.ro; 3 Bachelor Program in Nutrition and Dietetics, “Iuliu Hatieganu” University of Medicine and Pharmacy, 400337 Cluj-Napoca, Romania; raresadelin99@gmail.com; 4 Department of Internal Medicine/Gastroenterology, Faculty of Medicine, “Iuliu Hatieganu” University of Medicine and Pharmacy, 400337 Cluj-Napoca, Romania; catinean@gmail.com; 5 Department of Pharmaceutical Botany, “Iuliu Hatieganu” University of Medicine and Pharmacy, 23 Marinescu Street, 400337 Cluj-Napoca, Romania; mocan.andrei@umfcluj.ro

**Keywords:** psoriasis, microbiota, dietary approaches, probiotics, prebiotics

## Abstract

Psoriasis is a chronic inflammatory skin disease with autoimmune pathogenic characteristics and is caused by chronic inflammation, which results in uncontrolled keratinocyte growth and defective differentiation. The link between the gut microbiota and immune system regulation opened a novel angle to understand the pathogenesis of many chronic multifactorial diseases, including psoriasis. Current evidence suggests that modulation of the gut microbiota, both through dietary approaches and through supplementation with probiotics and prebiotics, could represent a novel therapeutic approach. The present work aims to highlight the latest scientific evidence regarding the microbiome alterations of psoriatic patients, as well as state of the art insights in terms of microbiome-targeted therapies as promising preventive and therapeutic tools for psoriasis.

## 1. Introduction

Psoriasis is a chronic inflammatory skin disease with autoimmune pathogenic characteristics and a solid hereditary susceptibility. The majority of psoriasis cases include chronic plaque-type psoriasis (known as *Psoriasis Vulgaris*). Typical clinical symptoms are sharply defined, erythematous, pruritic plaques. These can cover large areas of skin as they consolidate. The scalp, trunk, and extensor surfaces of the limb are the common sites [1]. Psoriasis is caused by chronic inflammation, which results in uncontrolled keratinocyte growth and defective differentiation. Epidermal hyperplasia coexists with inflammatory infiltrates constituted of dermal dendritic cells, macrophages, T lymphocytes, and neutrophils in the histology of psoriasis plaques [2].

The gut microbiota is represented by trillions of microorganisms that colonize the gastrointestinal tract and are involved in many local and systemic processes [3,4]. These microorganisms are bacteria, viruses, and eukaryotic species, and 90% of them belong to *Bacteroidetes* and *Firmicutes* phyla, followed by *Fusobacteria*, *Proteobacteria*, *Tenericutes*, *Actinobacteria*, and *Verrucomicrobia* [5].

Many factors can influence intestinal microbiota composition and functions, including dietary patterns, antibiotics, as well as the mode of delivery at birth having an essential role in the bacterial diversity [6,7,8].

Commensal bacteria, especially bacteria in the gut, contribute to maintaining a healthy immune system [9]. The intestinal mucosa host’s key immune system signaling molecules and cells, such as subpopulations of T cells, neutrophils, natural killer lymphocytes and macrophages, are sensitive to the microbial composition. Dysbiosis, a condition associated with the loss of beneficial microbial composition, as well as an overgrowth of pathogenic microbes, can have a direct impact on gut immune cells [10]. Short-chain fatty acids (SCFAs), such as propionate, acetate, and butyrate, are the end products of dietary fibers digested by gut microbiome components, with the potential to lower proinflammatory cytokine and chemokine production, suppressing inflammatory T cell function, and leading to a systemic anti-inflammatory effect in the body [11]. In contrast, lipopolysaccharides (LPS), which represent an element of the outer membrane of Gram-negative bacteria, could induce the over-expression of pro-inflammatory cytokines, such as tumor necrosis factor (TNF)-α, interleukin (IL)-6 and IL-8, promoting a moderate inflammation status in the body [12,13].

Numerous studies associate gastrointestinal health to skin homeostasis, with both the composition and function of the gut microbiota being disrupted in psoriasis patients [14,15]. The link between the gut microbiota and immune system regulation opened a novel angle to understand the pathogenesis of many chronic multifactorial diseases, including psoriasis. The present work aims to highlight the latest scientific evidence regarding the microbiome alterations of psoriatic patients, as well as state of the art insights in terms of microbiome-targeted therapies as promising preventive and therapeutic tools for psoriasis.

## 2. Gut Microbiota—An Overview

The gut microbiota is represented by a diverse collection of microorganisms found in the digestive systems of humans, and when compared to other sections of the body, it has the most significant number of microorganisms and the highest number of species [16]. This collection primarily consists of bacteria, but viruses and other eukaryotes invade the gastrointestinal tract shortly after birth [17,18]. The human gut microbiota begins to develop in the perinatal stage and is crucial to the regular functioning of the host organism [19]. It can produce several metabolic products when interacting with the host, positively or negatively impacting human health. The disruption of short-chain fatty acid production could have a variety of pathogenic repercussions for the host [20]. Moreover, patients with psoriatic arthritis [21] and multiple sclerosis (MS) have negatively changed SCFA levels, and the SCFAs could potentially affect the course and development of these diseases [22].

### The Implications of Diet and External Factors on the Composition of the Gut Microbiota

Diet is appreciated as the most potent modulator of both microbiota composition and function. It was revealed that dietary components have a beneficial impact on the host’s microbiota diversity [23]. Both vegetarian and low-calorie Mediterranean diets, rich in whole grains, probiotic foods, fruits and vegetables and bioactive dietary components, have been demonstrated to positively alter the host’s microbiota diversity; however, it is vital to note that both diets should persist longer than three months [24]. In contrast, a Western diet consisting of energy-dense, processed foods, high in fat and sugar, and low in fiber, decreased the diversity of the cecal microbiota, shifted its composition toward a pro-inflammatory profile by enhancing the *Desulfovibrionaceae* and *Proteobacteria’s* relative abundance, and altered the cecal metabolome [25]. Food additives frequently used in a Western diet, such as polysorbate 80 and carboxymethylcellulose, may disrupt gut homeostasis, contributing to tissue-damaging inflammatory responses [26]. Moreover, maltodextrin, a polysaccharide, causes endoplasmic reticulum stress in intestinal cells, decreasing mucus secretion and increasing the level of inflammation in mice models [27].

**Figure 1:**
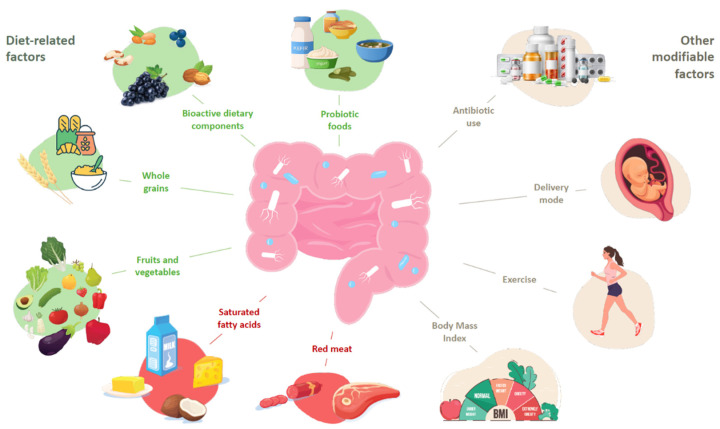
Factors associated with gut microbiota composition.

As presented in Figure 1, it is not only diet that impacts the gut microbiota. The physical activity and body mass index, mode of delivery and feeding in newborns, as well as the use of specific medication, especially antibiotics, are external factors that can change the composition of the intestinal microbiota [28,29,30,31,32]. Thus, in order to prevent such pro-inflammatory profiles and dysbiosis in the intestinal microbiota, it is essential to make dietary choices and lifestyle changes that are associated with better outcomes in gut microbiota diversity and functions [33,34].

## 3. Gut microbiome Alterations in Psoriasis

### 3.1. The Role of the Gut Microbiota in the Pathogenesis of Psoriasis

**Figure 2:**
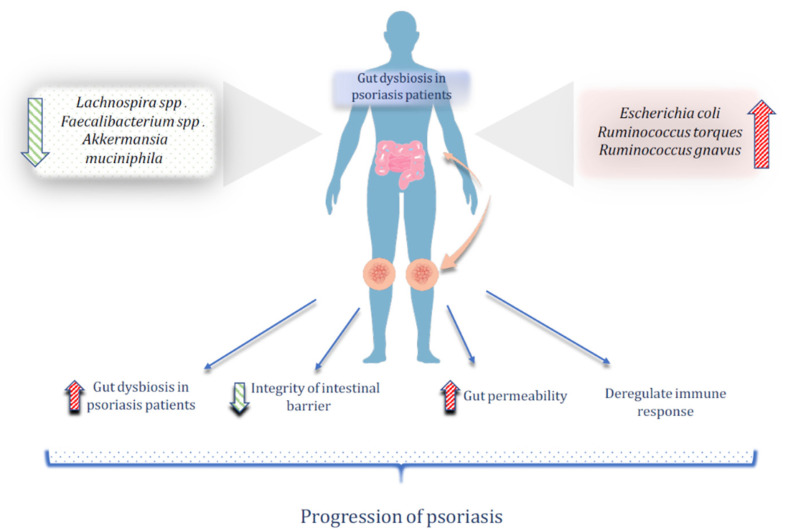
The role of gut dysbiosis in the pathogenesis of psoriasis. The green arrow represents the low diversity of potentially beneficial bacteria in the gut microbiota of psoriasis patients. The red arrow represents the high diversity of potentially harmful bacteria in the gut microbiota of psoriasis patients.

The gut microbiome’s diversity can have a significant impact on immunological development and disease risk, especially for autoimmune conditions, such as psoriasis [35]. Intestinal microbiota dysbiosis in psoriasis patients depends on the severity and status of the disease (Figure 2). Various studies show different results regarding the composition of the intestinal microbiota in psoriasis subjects. For example, levels of *Prevotella* spp. (species) were either higher [36] or lower [37] among psoriasis subjects compared to the healthy control. In both cases, intestinal dysbiosis was noticed. According to a study based on microbiota and inflammation-related variables, microbiota dysbiosis may produce an aberrant immune response in psoriasis. The microbiome changes were correlated with the degree of inflammation-related markers that were irregular in psoriasis patients, specifically the IL-2 receptor, which exhibited a positive relationship with *Phascolarctobacterium* and a negative relationship with *Dialister*. *Phascolarctobacterium* and *Dialister* relative abundances could be used as predictors of the psoriasis activity [38]. Moreover, complement 3 has a negative correlation with *Escherichia* level [38], which tends to be higher in psoriasis patients [39]. According to a study in Brazil that investigated the composition and diversity of the gut microbiota in 21 subjects with psoriasis, when compared to the control group, the psoriasis group showed a decrease in the *Lachnospira* and *Akkermansia muciniphila* species [40]. This decrease in *Akkermansia muciniphila* was also highlighted by another study that used 16S rDNA sequencing technology to examine microbiota composition in 14 psoriasis patients [41]. Such changes were linked to butanoate metabolism and butyrate production in the human colonic microbiota [42,43]. Butyrate has been implicated in the regulation of various inflammatory factors, including lipopolysaccharides, TNF-α, IL-10, IL-1β [44].

*Faecalibacerium* spp. showed a lower abundance in psoriasis patients with a lower richness and a difference in β diversity community composition [39,45], while *Ruminococcus torques* and *Ruminococcus gnavus* exhibited a greater abundance [45]. Although the number of subjects included in such investigations is limited, it appears that psoriasis patients have decreased functional potential in the gut microbiota, due to intestinal dysbiosis [45]. More investigation on the gut microbiota profile in psoriasis patients would bring a more reasonable perspective to the scientific field regarding the role of gut dysbiosis in the pathogenesis of psoriasis.

The immunological and inflammatory responses in psoriasis patients are affected by gut microbiota dysbiosis, enhanced pathways, and dysregulated metabolites [46].

Earlier, the identification of the IL-23/Th17 (T helper cells) axis as a significant signaling channel elucidated the mechanism of increased inflammation in psoriasis, in addition to the critical function of autoreactive T cells and cytokines [47]. Dendritic cells increase the proliferation of T lymphocytes in psoriasis, specifically T helper Th17 and Th22 in the acute phase and interferon-producing T cells in the chronic phase. T-cell infiltration in active psoriatic skin creates a cytokine environment, mandating individual gene profiles in keratinocytes. This could overexpress various inflammatory mediators, enhancing local immune reactivity [48,49]. Moreover, better outcomes of trials addressing TNF-α inhibitors as a treatment in psoriasis patients reflect the critical role of this cytokine in the immunopathogenesis of psoriasis [50,51]. TNF-α seems to have a modulatory role in the activation and production of cytokines by Th1 and Th17 cells [52]. Furthermore, NFKB1 (nuclear factor kappa B subunit 1) was shown to be elevated in psoriasis patients, exacerbating the symptoms of this condition. In particular, NFKB1 performs a crucial influence on keratinocytes in psoriasis by promoting Th1 and Th17 activation [53]. Additionally, overexpression of NFKB1 in psoriasis mice led to more pustules, an evident increase in acanthosis, as well as greater parakeratosis and desquamation [53].

The gut microbiota presents an essential role in host homoeostasis and immune response, particularly in Th17 cells [54]. For example, according to the findings of a recent prospective, randomized trial, dietary treatments can cause solid and repeatable changes in the immune system, suggesting that they have the potential to enhance immunological status as well as gut microbiome function. Fermented food intake decreased 19 cytokines, chemokines and other inflammatory serum proteins, including IL-6, IL-10, IL-12b [9]. Regarding IL-6, it was shown to be a predominant upstream signal for keratinocytes in mice with psoriasis-like dermatitis [55]. Curiously, adipocyte dysfunction was linked to metabolic syndrome and is related to an increase in the M1 macrophage population inside the adipose tissue. This could contribute to increased adipose tissue release of IL-6 and other pro-inflammatory cytokines that could subsequently promote insulin resistance via a variety of cellular signaling pathways, including mTOR and protein kinase C [56].

### 3.2. Changes in Gut Microbiota after Antipsoriatic Treatment

Biologic therapy in other inflammatory pathologies, for instance inflammatory bowel disease, might have a negative impact on the patients’ gut microbiota [57]. According to a transdisciplinary study published in “*Arthritis & Rheumatology*”, the use of an IL-17A inhibitor occurred in gut microbial dysbiosis and characteristics of subacute intestinal inflammation in a group of psoriatic arthritis and spondyloarthritis patients [58]. However, to date, only a few studies have investigated the changes in gut microbiota after antipsoriatic treatment.

### 3.3. TNF-α Inhibitor

Adalimumab (ADA), a TNF-α antagonist, was shown to be effective in the treatment of psoriasis, despite its possible adverse reactions [59]. Lihong Zhao et al. investigated the efficacy and safety of adalimumab in the treatment of psoriasis, as well as its impact on the gut microbiota. They evaluated changes in the pre-treatment and post-treatment intestinal microbiome composition in psoriasis patients following ADA medication and variations in the microbiome composition between psoriasis patients and healthy controls. The results showed no significant changes in the intestinal microbiome of patients before and after three months of ADA treatment [60]. Given the modest number of psoriasis participants included in this study (*n* = 13), long-term follow-up of patients treated with ADA and subsequent psoriasis research based on microbiota may provide further support for psoriasis treatment.

### 3.4. IL-17 and IL-12/23 Blockers

IL-17 inhibitor (secukinumab) and IL-12/23 inhibitor (ustekinumab) have already been proven to be effective in the treatment of moderate-to-severe psoriasis [61].

By addressing their effect on the gut microbiota, they were investigated in an observational and longitudinal study that gathered 114 fecal samples from 12 healthy controls and 34 psoriasis patients at baseline, 3 and 6 months following secukinumab or ustekinumab treatment. On the one hand, secukinumab therapy seemed to alter the gut microbiota more significantly than ustekinumab treatment, including increases in the relative abundance of the phylum *Proteobacteria* and decreases in *Bacteroidetes* and *Firmicutes*. Following secukinumab medication, the relative abundance of the families *Pseudomonadaceae*, *Enterobacteriaceae*, and *Pseudomonadales* increased considerably. On the other hand, there was no significant change in gut microbiome composition after ustekinumab treatment, and only the genus *Coprococcus* grew considerably after six months of ustekinumab therapy [62].

Furthermore, biologic therapy in psoriasis patients may impact the composition of the gut microbiota. In ten patients receiving systemic biologic therapy, six patients receiving anti-TNF-α (five receiving adalimumab and one receiving golimumab) and four receiving anti-IL-12/23 (ustekinumab), as well as 20 patients who had not received antipsoriatic systemic therapies in the previous six months or topical corticosteroids, α and β diversity vary dramatically. Bacterial biodiversity was found to be lower in the group of treated patients compared to the group of untreated patients. At the species level, treated subjects had significantly higher abundances of *Bacteroides plebeius*, *Roseburia faecis*, and *Bifidobacterium adolescentis*, and significantly lower abundances of *Bacteroides caccae*, *eggerthii* and *coprophilus*, *Blautia obeum*, *Alistipes indistinctus* and *massiliensis*, *Ruminococcus lactaris*, *Haemophilus parainfluenzae*. The species *Akkermansia muciniphila* showed the highest variation in relative abundance between treated and untreated individuals [63].

Nevertheless, more research on the influence of antipsoriatic medication on the intestinal microbiota of psoriasis patients, with a greater number of participants, is needed to better understand this topic.

## 4. Gut Microbiome-Targeted Therapies for Psoriasis

Current evidence suggests that modulation of the gut microbiota, both through dietary approaches and through supplementation with probiotics and prebiotics, could represent a new therapeutic target in autoimmune pathologies, for instance multiple sclerosis [64], celiac disease [65] and psoriasis [66]. In the following, we will discuss if the gut microbiota-targeted therapies, including dietary approaches and supplementation with bioactive dietary components, probiotics and prebiotics, could present health benefits in psoriasis patients.

### 4.1. Dietary Approaches

#### 4.1.1. Mediterranean Diet

The Mediterranean diet encourages a high consumption of plant-based foods, such as fruits, vegetables, nuts, legumes, grains and olive oil, while reducing the intake of red meat, dairy products, and processed products [67]. Recent evidence suggests that adherence to the Mediterranean diet could also impact the inflammatory markers in autoimmune diseases [68] and may reduce the severity status of certain dermatological pathologies [69,70]. For instance, adherence to the Mediterranean diet, specifically the use of extra virgin olive oil as the primary fat in the diet, reduced the disease severity among patients with suppurative hidradenitis by reducing the Hurley stage and the severity score [71]. Moreover, adherence to the Mediterranean diet seems to be negatively associated with the severity of acne; thus, increasing the consumption of foods specific to the Mediterranean diet can benefit people with acne [70,72].

On the one hand, the anti-inflammatory effects of a Mediterranean diet could be explained due to a high intake omega 3 fatty acids present in the Mediterranean diet that were linked with favorable outcomes regarding their effects in psoriasis patients [73]. On the other hand, the Mediterranean diet could also enrich the gut microbiota diversity, including bacteria with anti-inflammatory properties [74]. The anti-inflammatory effects of the Mediterranean diet in psoriasis patients were discussed in a prospective questionnaire study performed by Céline Phan et al. [75]. It was revealed that low adherence to the Mediterranean diet was correlated with a more severe status in psoriasis patients; however, this study did not approach the gut microbiota’s role in the anti-inflammatory effects observed in psoriasis patients but more on the biologically active components present in the Mediterranean diet. The same results, with the same perspective, were also supported by a cross-sectional study in 2015, with a smaller sample of mild-to-severe psoriasis patients (*n* = 62), which can represent a limitation of the study [76]. The results showed that the PASI (Psoriasis Area and Severity Index) score, measured for the severity status of psoriasis, presented a significant association with the percentage of the C-reactive protein levels, which was negatively correlated with adherence to the Mediterranean diet. The fish and extra virgin olive oil intake were both independent predictors of PASI score and C-reactive protein levels [76]. Similarly, an energy-restricted diet intended to enhance the intake of omega-3 and decrease omega-6 PUFAs improved the metabolic profile and increased the responsiveness to immunomodulating treatment in obese psoriatic patients [73]. Regardless of whether the role of the diet in modulating the gut microbiota in psoriasis patients is not fully understood, considering that psoriasis patients usually tend to have a hypercaloric diet rich in processed foods, saturated fats, sugar and sodium with low nutritional quality and a high inflammation profile [77], it is essential to consider modifying dietary habits among these patients as an adjuvant therapy to the immunomodulating treatment.

#### 4.1.2. Gluten-Free and Low-FODMAP Diet

Recent evidence shows that psoriasis corelates with celiac disease [78] and that patients with psoriasis present a higher risk of developing this autoimmune disease [79]. Thus, psoriasis patients must benefit from screening for celiac disease for a more precise and effective nutritional therapy regarding gastrointestinal and inflammatory symptoms.

Despite the fact that a gluten-free diet was previously linked with favorable outcomes in clinical studies, including patients suffering from other diseases, for instance autoimmune thyroid in women [80] or type 1 diabetes with subclinical celiac disease [81], at times, insufficient studies address the efficacy of such a diet in psoriasis patients without celiac disease. In fact, dietary gluten intake is not considered a risk factor for psoriasis or psoriatic arthritis [82]. Moreover, the National Psoriasis Foundation from the United States performed a systematic review addressing the dietary recommendation for adults with psoriasis or psoriatic arthritis [83]. They only recommend a gluten-free diet to psoriasis patients who have been diagnosed with celiac disease. They advocate a 3-month gluten-free diet trial for psoriasis patients with gluten sensitivity as an adjuvant intervention to the regular treatment, but for patients without such symptoms, a gluten-free diet was not indicated, due to limited data about this subject. However, in another national survey from the United States with 1206 subjects, psoriasis patients reported skin improvement after reducing the intake of alcohol and gluten and after increasing their intake of fish oil and vegetables [84].

Short-chain carbohydrates and sugar alcohols are restricted in the low FODMAP (which stands for fermentable, oligosaccharides, disaccharides, monosaccharides, and polyols) diet. The restriction of these dietary components has been demonstrated to be beneficial in individuals with irritable bowel syndrome by significantly reducing the abdominal pain and bloating [85], but limited studies have addressed the effectiveness of a low-FODMAP diet in psoriasis patients. In mice, the reduction in dietary FODMAPs did not increase nor reduce inflammation. Moreover, it seems as if the microbiota profile changes were caused by inflammation rather than diet, and a low FODMAP intake resulted in proteolytic fermentation following inflammation [86]. Results from a randomized clinical trial that studied the effects of a low-FODMAP diet on fecal microbiome and inflammatory markers in patients with inflammatory bowel disease presented a reduction in the fecal abundance of *Bifidobacterium adolescentis*, *Bifidobacterium longum*, and *Faecalibacterium prausnitzii*, but no differences were observed with the inflammatory markers [87]. Although a gluten-free and low-FODMAP diet appears to be beneficial in managing the gastrointestinal symptoms and modulating the gut microbiota in patients with irritable bowel syndrome [88], further research is required to determine the long-term efficacy and safety of such a dietary intervention on nutritional adequacy and the gut microbiome of psoriasis patients.

### 4.2. Probiotics/Prebiotics/Synbiotics

Probiotics are living microorganisms that can be found in fermented foods or nutritional supplements and provide beneficial health properties to the host when they are ingested or administered [89]. Probiotics enclose a wide range of microorganisms. Bacteria from the *Lactobacillaceae* and *Bifidobacteriaceae* families are the most frequent, but other bacteria, as well as yeasts, can be administered as probiotics [89,90]. In addition to probiotics, nondigestible dietary components, such as fructooligosaccharides (FOS), inulins, or galactooligosaccharides, promote the development of beneficial bacteria in the intestinal microbiota, and they are named prebiotics [91]. When ingested together from the same mixture or dietary supplement, they are called synbiotics [92].

The revelation of the gut microbiota’s function in inflammatory diseases opens the door to therapeutic microbiome modulation [93]. Probiotic and prebiotic supplementation might be employed as a novel therapeutic in the treatment and prevention of a variety of skin conditions [94,95].

The therapeutic approach of probiotic/prebiotic/synbiotic supplementation among psoriasis patients has begun to arouse the interest of many researchers; thus, at the moment, several studies are addressing this issue in both experimental and clinical studies.

In imiquimod-induced psoriasis-like mice, the supplementation with probiotics for two weeks resulted in great relief from psoriasis-like pathological characteristics [96]. More precisely, *Bifidobacterium adolescentis* CCFM667, *B*. *breve* CCFM1078, *Lactobacillus paracasei* CCFM1074, and *L*. *reuteri* CCFM1132 successfully reduced erythema, scaling, and thickening, but *B. animalis* CCFM1148, *L. paracasei* CCFM1147, and *L. reuteri* CCFM1040 showed modest effects. Moreover, the immune responses through the IL-23/Th17 axis, *B. adolescentis* CCFM667, *B. breve* CCFM1078, *L. paracasei* CCFM1074, and *L. reuteri* CCFM1132 were beneficial in alleviating psoriasis by suppressing the cytokine activity. The strains that effectively treated psoriasis symptoms elevated acetate or propionate levels in the gut microbiota. The levels of acetate were considerably inversely connected to IL-17 and IL-23, whereas the levels of propionate were significantly inversely related to the levels of IL-23. This could demonstrate the practical applicability of probiotic supplementation in regulating inflammation levels among psoriasis patients [96].

Promising outcomes regarding the efficacy and safety of oral administration of probiotic strains/prebiotics/synbiotics in psoriasis patients were also highlighted by clinical studies. A case report from 2012 showed the benefits of a *Lactobacillus* probiotic administration, one sachet thrice daily with biotin 10 mg once daily, in the case of a 47-year-old woman who had psoriasis with pustules all over her body and did not respond to the anti-psoriatic treatment. After fifteen days of supplementation, no new lesions appeared, and the ones existing started involuting. After six months of supplementation, the subject was free of lesions [97].

Groeger David et al. showed in 2013 that the immunomodulatory effects of the microbiota in humans are not limited to the mucosal immune system but extend to the systemic immune system. The authors performed a study that revealed the beneficial effects of *Bifidobacterium infantis* 35,624 in psoriasis patients not receiving anti-psoriatic treatment. The supplementation for 6–8 weeks resulted in reduced pro-inflammatory status by lowering the plasma CRP and LPS-stimulated TNF-α and IL-6 levels [98].

A recent randomized, double-blind trial performed by Jalal Moludi et al. [66] showed that the supplementation with *Lactobacillus* strains in fifty psoriasis patients for eight weeks improved the quality of life and the inflammatory markers. Compared with the placebo group, a significant reduction in PASI and psoriasis symptom scale was found in psoriasis patients. Moreover, the total antioxidant capacity levels were increased, while a decrease in C-reactive protein was identified in the intervention group. However, it is not mentioned if the subjects received anti-psoriatic treatment previous to the study. Jalal Moludi et al. also highlighted the improvement in PASI score and quality of life among psoriasis patients by assessing the efficacy of a multi-strain probiotic in forty-six subjects. Besides an improvement in PASI score and quality of life, after two months of supplementation, the blood pressure, pro-inflammatory cytokines (hs-CRP and IL1-β), and LPS serum levels were considerably reduced [99].

Chuhui Lin et al. investigated the effect of Bacteroides fragilis BF839 in 26 psoriasis patients. The subjects received the probiotic for 12 weeks while maintaining the anti-psoriatic treatment. The results showed a statistically significant difference (*p* < 0.01) in the reduction in PASI score, with only one case of constipation as a side effect [100]. Unfortunately, the changes in the composition of the intestinal microbiota among patients with psoriasis have not been measured, which can represent a limitation for these studies.

Another twelve-week randomized, double-blind and placebo-controlled trial was performed to assess the therapeutic efficacy and safety of *Bifidobacterium longum* CECT 7347, *B. lactis* CECT 8145, and *Lactobacillus rhamnosus* CECT 8361 in ninety psoriasis patients receiving anti-psoriatic treatment (topical corticosteroid betamethasone in combination with calcipotriol) [101]. Besides a reduction in PASI score, a complete loss of the genera *Micromonospora* and *Rhodococcus* and an increase in *Collinsella* and *Lactobacillus* were discovered in the probiotic group. However, it is difficult to confirm the impact of probiotic supplementation separated from topical treatment or whether the treatment had any impact on the gut microbiota changes. Curiously, a lower abundance of *Collinsella* genera was linked with other autoimmune disorders [102,103] and with lower production of butyrate in the intestinal microbiota [104].

In lipopolysaccharide-induced endotoxemic mice, the supplementation with prebiotics inulin, xylan and polysaccharides regulated key mediators, such as IL-18, and IL-22 and suppressed the inflammatory Th cell response in the ileum [105]. Moreover, the gut microbiota composition changed significantly in obese mice supplemented with cellulose, short-chain FOS and inulin for four weeks. Mice fed short-chain FOS presented the highest abundance in *Actinobacteria* and *Verrucomicrobia*, specifically *Akkermansia* spp. [106]. Such modifications were also observed in the gut microbiota of psoriasis models (*Traf3ip2* mice) after the supplementation with fucoidan, a dietary seaweed fiber. The relative proportions of *Bacteroidetes* and *Proteobacteria* increased considerably in the fucoidan diet group’s fecal microbiota at the phylum level. The genera *Coprococcus*, unclassified members of the *Ruminococcaceae* family, and unclassified members of the order *Clostridiales* were lower in the fecal microbiota of the intervention group. Moreover, a decrease in facial scratching and ameliorated psoriasis symptoms were also observed, among an increased mucin volume in feces [107].

Very few studies have investigated the effect of prebiotics or symbiotics among patients with psoriasis. For instance, a randomized, double-blind controlled clinical trial evaluated the efficacy of a synbiotic, including *Lactobacillus casei*, *L*. *acidophilus*, *L*. *rhamnosus*, *L*. *bulgaricus*, *Bifidobacterium breve*, *B*. *longum*, *Streptococcus thermophiles* and FOS, on the serum electrolyte levels in psoriasis patients. The result highlighted that Fe, Ca, Mg, P, Zn, and Na levels were greater at week twelve compared to the baseline in psoriasis patients. The authors concluded that such changes might occur due to an improvement in mineral absorption by favorable effects on the gastrointestinal system [108]. However, no changes in the gut microbiota were examined in this study. Consequently, while recent studies suggest encouraging effects of probiotic/prebiotic supplementation among psoriasis patients, further research with a more significant number of subjects and various bacterial strains and prebiotics is required for a more effective therapeutic nutritional strategy in those patients.

### 4.3. Bioactive Dietary Components

Non-essential biomolecules that are present in foods or dietary supplements (e.g., polyphenols, glucosinolates, curcumin, omega-3 polyunsaturated fatty acid) can alter metabolic processes in the body and were shown to provide health benefits in many pathological conditions, including gut microbiota dysbiosis [109,110,111,112]. Recent studies show promising results regarding the efficacy of bioactive dietary components in autoimmune diseases, even if the mechanism of action is not fully understood [113].

The efficacy of bioactive dietary components in psoriasis patients was also questioned in several clinical trials, but none of them correlated these changes with the gut microbiota. Phenolic compounds, which have antioxidant, anti-inflammatory, and immunomodulatory properties, have been related to the beneficial properties in immune-mediated inflammatory diseases [114]. For instance, after three months of treatment with 500 mg of an olive polyphenolic extract, the PASI score significantly decreased, with 25% in the psoriasis group receiving the supplement [115]. Accordingly, the polyphenolic extract from *Abies alba* improved psoriasis patients’ signs and symptoms by lowering the IL-1β production; however, the improvement was not significant. As previously found, a diet rich in polyphenols and polyunsaturated fatty acids was linked with modified gut microbiota composition [116]. Precisely, significantly increased microbial diversity was noticed with an increased number of *Bifidobacteria*. Lower diversity of *Bifidobacteria* is known to be associated with systemic inflammation and immune dysregulation of intestinal Th2 and Th17 cytokines [117].

#### 4.3.1. Curcumin

Curcumin, a natural compound known for its anti-inflammatory activity, accumulates in the gastrointestinal tract following oral administration and may exercise its regulatory effect by modulating the microbial diversity and composition of the intestinal microflora [118]. For instance, changes in gut microbiota after curcumin supplementation were highlighted by a human randomized placebo-controlled trial that studied the impact of turmeric and curcumin dietary supplementation in 30 healthy subjects. The supplementation group received 6000 mg of *Curcuma longa* extract daily and the microbiota analyses were performed at the beginning of therapy and after 8 weeks. All of the participants had substantial changes in microbiota composition over time, as well as a personalized response to therapy. Most *Clostridium* spp., *Bacteroides* spp., *Citrobacter* spp., *Cronobacter* spp., *Enterobacter* spp., *Enterococcus* spp., *Klebsiella* spp., *Parabacteroides* spp., and *Pseudomonas* spp. were uniformly increased in the responsive participants. The lower relative abundance of many *Blautia* spp. and the majority of *Ruminococcus* spp. were exhibited in both groups [119]. Furthermore, curcumin was demonstrated to be efficient for inducing mucosal immune cells with regulatory features in mice by significantly suppressing NFKB activation in the colonic epithelium and controlling the production of inflammatory mediators [120]. Furthermore, the number of butyrate-producing bacteria and fecal butyrate levels increased, as did the proliferation of CD4+ Foxp3+ regulatory T cells and CD103+ CD8- regulatory dendritic cells [120]. The oral supplementation with curcumin in psoriasis patients was evaluated for twelve weeks, resulting in a significant reduction in PASI score with a decrease in IL-22 serum levels [121]. Interestingly, in the gut microbiota of chronic kidney disease patients, curcumin supplementation was linked with lower *Escherichia* spp. and *Shigella* spp., and a greater abundance of *Lachnoclostridium*. Besides these changes, lower plasma levels of pro-inflammatory mediators (CCL-2, IFN-γ, and IL-4), as well as lipid peroxidation, were also reported [122]. Moreover, the oral administration of curcumin at the same time with local phototherapy in patients with plaque psoriasis seems to induce a quicker and more progressive therapeutic response to the treatment [123]. Considering that these inflammatory pathogenetic mechanisms are similar to those found in psoriasis subjects [9,53], curcumin supplementation could represent a future perspective regarding the management of this pathology.

#### 4.3.2. Omega-3 Fatty Acids

There is a link showing the influence of PUFAs on immunity via modulating the gut microbiota. For instance, the administration of flaxseed oil in rats resulted in a higher level of SCFA production and a better microbial diversity, with *Lactobacillus*, *Firmicutes*, *Butyrovibrio*, and *Bifidobacterium* being negatively linked with pro-inflammatory markers (IL-1β, IL-6, IL-10, IL-17A, and TNF-α) [124].

Regarding the efficacy of omega-3 fatty acids in psoriasis, a recent randomized controlled trial performed by Kåre Steinar Tveit et al. [125] highlighted that supplementation with herring roe oil (containing 292 mg of polyunsaturated fatty acids omega-3) leads to a significant improvement in the PASI score in psoriasis subjects. However, no significant changes were observed at the levels of inflammatory markers [125]. Another 6-week randomized clinical trial, which included healthy subjects, highlighted that a daily dose of 500 mg omega-3 increased the *Coprococcus* spp. and *Bacteroides* spp. and significantly decreased *Collinsella* spp. At the same time, serum levels of iso-butyrate and isovalerate seemed to increase by the end of the study [126]. Curiously, high levels of *Collinsella* spp. characterize the fecal microbiota of psoriasis subjects [37], while SCFAs and branched SCFAs, such as iso-butyrate and isovalerate, are known for their anti-inflammatory effects [127]. Moreover, omega-3 PUFAs, which interfere with the synthesis of pro-inflammatory eicosanoids [128], suppress the transcription of inflammatory cytokines via inhibiting NFKB-mediated inflammation [129], which is similar to the processes underlying inflammation in psoriasis [53]. Therefore, omega-3 supplementation may benefit people diagnosed with *psoriasis vulgaris* in terms of regulating the pathophysiological process of inflammation via modulating the gut microbiota.

#### 4.3.3. Resveratrol

Resveratrol is a nonflavonoid polyphenol compound found in plants and is essential for its anti-inflammatory benefits [130]. More studies investigated the effect of resveratrol on the gut microbiota, showing promising results regarding the biodiversity and microbial composition, as well as an improved intestinal barrier function and a greater intestinal permeability [131,132,133]. For instance, in piglets, dietary supplementation of 300 mg/kg of resveratrol for 28 days led to an anti-inflammatory effect by down-regulating toll-like-receptor 4 mRNA in the intestine and lowering the release of critical inflammatory compounds (IL-1β, TNF-α), as well as increasing the secretion of immunoglobulin [134]. Favorable effects of resveratrol on the intestinal microbiota have also been found in mice experiments. Oral administration of resveratrol is able to enhance intestinal barrier function, while also reducing permeability and inflammation. The composition of the gut microbiota was drastically changed following resveratrol treatment. Resveratrol therapy restored dysbiosis in mice by increasing the abundance levels of *Bacteroides*, *Alistipes*, *Rikenella*, *Odoribacter*, *Parabacteroides*, and *Alloprevotella* taxa, indicating a possible function for the microbiome [133]. Moreover, resveratrol administration, 400 mg/kg resveratrol for 8 weeks, increases the population of the butyrate producers *Blautia* and *Dorea* in the *Lachnospiraceae* family in high-fat diet-fed rats [135]. The enrichment of the *Lachnospiraceae* family was also highlighted by another study performed on high-fat diet-fed mice administrated with 300 mg/kg/day resveratrol for 16 weeks [136]. As previously presented, psoriasis patients tend to have lower levels of *Lachnospiraceae* family and *Blautia* compared with healthy individuals [40,137]; thus, resveratrol supplementation could lead to improvements in gut microbial diversity among these patients.

#### 4.3.4. Quercetin

Quercetin is a plant flavonol classified as a polyphenol flavonoid. It may be found in a wide range of fruits, vegetables, and leaves, seeds, and grains [138]. Many studies have previously shown the advantages of quercetin, especially regarding its anti-inflammatory, cytoprotective and immunosuppressive properties [139,140,141]. Recent research has begun to describe the influence of quercetin on the gut microbiota, due to an increased interest in this topic [131,142,143]. For instance, quercetin seems to ameliorate gut microbiota dysbiosis that drives hypothalamic damage and hepatic lipogenesis in monosodium glutamate-induced abdominal obesity mice. The quercetin therapy specifically reversed *Firmicutes* spp. and the *Firmicutes*/*Bacteroidetes* ratio was reduced following quercetin therapy. More than that, the authors confirmed a decrease in *Lachnospiraceae* and *Ruminicoccaceae* family*,* as well as an improvement in intestinal barrier function [144].

A recent study addressed the effect of quercetin supplementation (30, 60 and 120 mg/kg) on imiquimod-induced mice, showing drastically reduced PASI scores, lower temperature of psoriasis-like lesions, and improved psoriatic plaques. Furthermore, quercetin successfully reduced serum TNF-α, IL-6, and IL-17 levels strengthened the anti-inflammatory effect and reduced buildup in skin tissue produced by imiquimod in mice. The authors of the study concluded that this process might be linked to the regulation of the NFKB pathway [145]. Moreover, oral supplementation with quercetin, a dietary flavonoid extracted from *Fagopyrum tataricum,* reduced imiquimod-induced psoriasis-like dermatitis in mice, dramatically lowering keratinocyte proliferation and aberrant differentiation, as well as inflammatory cell infiltrates. A reduced expression of cytokines on the IL-23/Th17 axis and a reduced Th17 cell response was noticed after the oral administration of quercetin [146]. However, more research is needed to determine the exact relationship of quercetin with the gut microbiota and whether it may play a key role in modulating the gut microbiota among psoriasis patients.

As presented in Table 1, most studies evaluating the effects of diet and biologically active compounds on the intestinal microbiome are still in the preclinical phase. Table 2 summarizes most of the clinical trials that address the efficacy of such supplements in psoriasis with favorable results regarding PASI score, proinflammatory cytokine levels, and beneficial results on the quality of life of these patients. However, the positive intestinal modulation of psoriasis patients in the context of supplementation with probiotics, prebiotics, and biologically active compounds may play a key role in the fortunate clinical trial outcomes.

### 4.4. Fecal Microbiota Transplantation

Fecal microbiota transplantation (FMT) trials are more and more promising regarding the health benefits of inflammatory diseases. In 2015, Paul Moayyedi et al. showed that the FMT in active UC patients resulted in a greater microbial diversity along with the remission of the disease [148]. Promising results were also highlighted by Sudarshan Paramsothy et al. in an 8 week clinical trial. FMT increased gut microbial diversity and altered microbial composition by enhancing the *Eubacterium hallii* and *Roseburia inulivorans* species in active UC patients; all these changes along with the remission of the disease [149]. Moreover, FMT seems to be more efficient in treating *Clostridium difficile* infection compared with fidaxomicin [150]. The efficacy of FMT in psoriasis patients is still a research topic, but promising results from clinical trials have started to arouse interest. In a five week interventional clinical trial, a subject with plaque psoriasis and IBS was administrated FMT twice via endoscopy and colonoscopy. The body surface area, PASI score, dermatology life quality index, intestinal symptoms and serum level of TNF-α were all improved after the intervention with no adverse reactions observed [147]. No gut microbiota changes were measured, and the small number of subjects represents a limitation of the study. However, in peripheral psoriatic arthritis patients, FMT is not that efficient in treating the active disease [151]. Although FMT may bring some benefits regarding the severity of the disease in patients with psoriasis, more clinical trials are needed to demonstrate this and to investigate whether or not modulation of the intestinal microbiota plays a crucial role in this process.

## 5. Conclusions and Further Perspectives

This review highlighted the strong connection between psoriasis and the gut microbiota with the final purpose of adding novel wisdom for discovering the relationship between the altered gut microbiota in psoriasis patients, but there are still challenges and limitations that further research should address. Firstly, there is a need for improving protocols regarding collection, transportation, storage and DNA extraction in both animal and human studies to allow for optimal comparisons between studies. Moreover, there are insufficient data on the potential therapeutic approach to modulating the gut microbiota for better outcomes in psoriasis patients. Despite the increasing number of studies that highlight the microbial disruption in psoriatic patients, the data regarding microbiota modulation are lacking, meaning that the therapeutic strategy in clinical practice is based on evidence from other inflammatory and autoimmune pathologies, where the ability of diet, prebiotic/probiotic protocols, and biologically active compounds to modulate the gut microbiota have been demonstrated, as well as the therapy experience.Given that the severity and status of psoriasis are closely related to alterations in the intestinal microbiome, maintaining a balance in bacterial species using the aforementioned modulating factors could be an effective way to prevent the aggravation of the disease in these patients. Thus, additional human studies that include an accurate nutritional evaluation and therapeutic protocols are required in order to better understand the relationship between diet and microbiota in psoriasis patients. We anticipate that comprehensive study will soon enable us to characterize the gut microbiota as a tool for many diseases including psoriasis, and will allow lifestyle interventions and other dietary protocols to serve as cornerstones in treating the microbiome alteration of psoriatic patients.

## Figures and Tables

**Table 1:** The effects of low-FODMAP diet and biologically active compounds on the intestinal microbiome.

Therapy	Study Population	Intervention	Outcomes	Reference
Low-FODMAP diet	Crohn’s disease or ulcerative colitis patients Randomized *n* = 52 No previous probiotics, prebiotics, azathioprine, mercaptopurine, methotrexate, or biologics	Low-FODMAP diet for 4 weeks	↓*Bifidobacterium adolescentis*, ↓*Bifidobacterium longum*, ↓*Faecalibacterium prausnitzii*	Selina R. Cox et al. [87]
Omega-3 fatty acids	6-week-old female rats	1 mg/kg/day of flaxseed oil by gavage for 8 weeks	↑*Allobaculum*, ↑*Lactobacillus*, ↑*Butyrivibrio*, ↑*Desulfovibrio*, ↑*Bifidobacterium*, ↑*Faecalibacterium*, ↑*Parabacteroides* ↓*Actinobacteria*, ↓*Bacteroides*, ↓*Proteobacteria*, ↓*Streptococcus*, ↓*Firmicutes*/*Bacteroidetes* ratio	Ting Wang et al. [124]
Resveratrol	Diabetic nephropathy mice	Oral administration of 10 mg/kg/day resveratrol for 12 weeks	↑*Bacteroides*, ↑*Alistipes*, ↑*Rikenella*, ↑*Odoribacter*, ↑*Parabacteroides*, ↑*Alloprevotella*	Ting-Ting Cai et al. [133]
	High-fat diet-fed rats	400 mg/kg/day resveratrol, 200 mg/kg/day sinapic acid or both for 8 weeks	↑*Blauta* spp. ↑*Dorea* spp. ↓*Bacteroides* spp. ↓*Desulfovibrionaceae* spp.	ChenYang et al. [135]
	High-fat diet-fed mice	300 mg/kg/day resveratrol for 16 weeks	↑*Lachnospiraceae* family	Pan Wang et al. [136]
Quercetin	Monosodium glutamate-induced abdominal obese mice	5 mg/kg quercetin dissolved in 0.15% carboxymethylcellulose sodium, administrated by gavage for 6 weeks	↓*Firmicutes*/*Bacteroidetes* ratio ↓*Firmicutes* ↓*Bacteroides* spp. ↓*Lachnospiraceae* spp., ↓*Ruminicoccaceae* spp.	Lijun Zhao et al. [144]

↓—decreased, ↑—increased.

**Table 2:** The effect of probiotics, synbiotics and bioactive dietary components supplementation in human subjects.

Therapy	Study Population	Design	Intervention	Outcomes		Reference
Probiotics	47-year-old woman with psoriasis, having pustules all over her body; non-responsive to the anti-psoriatic treatment	6 month case report	*Lactobacillus* probiotic one sachet thrice daily with biotin 10 mg once daily for 6 months	In 15 days, the lesions started involuting; reduced blood sugar level After 6 months she was free of lesions		Metikurke Vijayashankar et al. [97]
	Psoriasis patients *n* = 26 PASI < 16 Healthy subjects *n* = 22 No previous immunosuppresant therapy	8 week RCCT ^1^	*Bifidobacterium infantis* 35,624	↓IL-6, ↓TNF-α, ↓CRP		Groeger David et al. [98]
	Psoriasis patients *n* = 50 Randomized	8 week RCCT	*Lactobacillus acidophilus*, *Bifidobacterium bifidum*, *Bifidobacterium lactis*, *Bifidobacterium langum* 1.8 × 10^9^ CFU/capsule	↑DLQI ^2^, ↑TAC ^3^, ↓PASI score, ↓PSS ^4^, ↓CRP, ↓IL-6		Jalal Moludi et al. [66]
	Psoriasis patients *n* = 46 Randomized	2 month RCCT	Probiotic capsules with multi-strain bacteria 1.6 × 10^9^ CFU/g	↑QOL ^5^, ↓serum LPS levels, ↓CRP, ↓IL-1β		Jalal Moludi et al. [99]
	Psoriasis patients *n* = 27 Received anti-psoriatic treatment	12 week single-arm, clinical trial	*Bifidobacterium fragilis* BF839	1 patient was excluded from the trial; ↓PASI score 1 case of side effect: constipation		Chuhui Lin et al. [100]
	Psoriasis patients receiving topical anti-psoriatic treatment, age 18–70, PASI > 6 *n* = 90, Randomized	12 week double-blind, RCCT	*Bifidobacterium* *longum* CECT 7347, *B. lactis* CECT 8145 and *Lactobacillus* *rhamnosus* CECT 8361 with a total of 1 × 10^9^ CFU/capsule	2 patients did not complete the study ↓PASI score; loss of the genera ↓*Micromonospora*, ↓*Rhodococcus*, ↑*Collinsella*, ↑*Lactobacillus*		Vicente Navarro-López et al. [101]
Synbiotic	Psoriasis patients *n* = 64	12 week double-blind RCCT	*Lactobacillus casei, L. acidophilus, L. rhamnosus, L. bulgaricus, Bifidobacterium breve, B. longum, Streptococcus thermophiles* and FOS	8 patients from the intervention group and 18 patients from the control discontinued the study; ↑ serum levels of Fe, Ca, Mg, P, and Zn due to favorable effects on the gastrointestinal system		Ali Akbarzadeh et al. [108]
Curcumin	Healthy human subjects *n* = 30 randomized No previous antibiotic, topical medication, or oral turmeric/curcuma supplement	8 week double-blind RCCT	Supplementation with 6000 mg/daily *Curcuma longa* extract	↑*Clostridium* spp., ↑*Bacteroides* spp., ↑*Citrobacter* spp. ↑*Cronobacter* spp. ↑*Enterobacter* spp., ↑*Enterococcus* spp., ↑*Klebsiella* spp., ↑*Parabacteroides* spp., ↑*Pseudomonas* spp., ↓ *Blautia* spp., ↓*Ruminococcus* spp.		Christine T. Peterson et al. [119]
	Psoriasis patients *n* = 63, PASI < 10. Randomized Receiving anti-psoriatic treatment	12 week double-blind RCCT	2 g/day of curcumin	↓PASI score, ↓ IL-22 serum levels		Emiliano Antiga et al. [121]
Omega 3 fatty acids	Psoriasis patients *n* = 64. Randomized PASI < 10 53% of subjects used local anti-psoriatic maintenance treatment	26 week double-blind RCCT	Herring roe oil (containing 292 mg of polyunsaturated fatty acids omega-3), Daily dose: 2,6 g EPA and DHA	6 patients from the interventional group did not complete the trial ↓PASI score No difference in inflammatory markers		Kåre Steinar Tveit et al. [125]
	Healthy subjects *n* = 69 Randomized No previous treatment	6 week randomized interventional trial	Daily dose of 500 mg of omega 3 (165 mg EPA, 110 mg DHA) vs. 20 g inulin	Inulin: ↑*Bifidobacterium* spp. ↑*Lachnospiraceae* spp. ↑iso-valerate ↑iso-butyrate ↑butyrate	Omega 3: ↑iso-valerate ↑iso-butyrate ↑ *Coprococcus* ↑ *Bacteroides* ↓*Colinsella*.	Amrita Vijay et al. [126]
Fecal microbiota transplantation	Severe plaque psoriasis and IBS patient *n* = 1	5 week interventional clinical trial	FMT upper endoscopy and colonoscopy	↓BSA ^6^, ↓PASI, ↑DLQI, ↓TNF-α Improved intestinal symptoms		G. Yin et al. [147]

^1^ RCCT = Randomized controlled clinical yrial; ^2^ DLQI = Dermatology Life Quality Index; ^3^ TAC = total antioxidant capacity; ^4^ PSS = Psoriasis Symptom Scale; ^5^ QOL = Quality of Life Index, ^6^ BSA = body surface area, ↓—decreased, ↑—increased.

## Data Availability

Not applicable.
